# Biotelemetric Monitoring of Brain Neurochemistry in Conscious Rats Using Microsensors and Biosensors

**DOI:** 10.3390/s90402511

**Published:** 2009-04-14

**Authors:** Giammario Calia, Gaia Rocchitta, Rossana Migheli, Giulia Puggioni, Ylenia Spissu, Gianfranco Bazzu, Vittorio Mazzarello, John P. Lowry, Robert D. O’Neill, Maria S. Desole, Pier A. Serra

**Affiliations:** 1 Department of Neuroscience, Medical School, University of Sassari, Viale S. Pietro 43/b, 07100 Sassari, Italy; E-Mails: gmcalia@uniss.it (G.C.); grocchitta@uniss.it (G.R.); rmigheli@uniss.it (R.M.); Giuliapmg@yahoo.it (G.P.); yspissu@uniss.it (Y.S.); gbazzu@uniss.it (G.B.); desole@uniss.it (M.-S.D.); 2 Department of Biomedical Sciences, Medical School, University of Sassari, Viale S. Pietro 43/b, 07100 Sassari, Italy; E-Mails: vmazza@uniss.it (V.M.);; 3 Department of Chemistry, National University of Ireland, Maynooth, Co. Kildare, Ireland; E-Mail: John.Lowry@nuim.ie (J.-P.L.);; 4 UCD School of Chemistry and Chemical Biology, University College Dublin, Belfield, Dublin 4, Ireland; E-Mail: robert.oneill@ucd.ie (R.-D.O.)

**Keywords:** Biotelemetry, microsensor, biosensor, glucose, oxygen, ascorbic acid

## Abstract

In this study we present the real-time monitoring of three key brain neurochemical species in conscious rats using implantable amperometric electrodes interfaced to a biotelemetric device. The new system, derived from a previous design, was coupled with carbon-based microsensors and a platinum-based biosensor for the detection of ascorbic acid (AA), O_2_ and glucose in the striatum of untethered, freely-moving rats. The miniaturized device consisted of a single-supply sensor driver, a current-to-voltage converter, a microcontroller and a miniaturized data transmitter. The redox currents were digitized to digital values by means of an analog-to-digital converter integrated in a peripheral interface controller (PIC), and sent to a personal computer by means of a miniaturized AM transmitter. The electronics were calibrated and tested *in vitro* under different experimental conditions and exhibited high stability, low power consumption and good linear response in the nanoampere current range. The *in-vivo* results confirmed previously published observations on striatal AA, oxygen and glucose dynamics recorded in tethered rats. This approach, based on simple and inexpensive components, could be used as a rapid and reliable model for studying the effects of different drugs on brain neurochemical systems.

## 1. Introduction

Details of the links between neurochemical and brain physiological functions or neurodegenerative diseases are mostly unknown. Because of its high energy metabolism, related to anatomical characteristics and physiology, the central nervous system (CNS) is assumed to be particularly sensitive to reactive oxygen species (ROS). Oxidative stress (OS) is crucial for the modulation of fundamental cellular functions such as apoptosis, calcium mobilization, and ion transport, all of which are involved in excitotoxicity. [1]. OS results from a disparity involving the physiological antioxidant capability and free radical synthesis [2]. Ascorbic acid (AA) is a water soluble vitamin that possesses radical scavenger properties against ROS [3], and represents the most important low molecular weight antioxidant in the brain. Even if not synthesized in humans, AA is an essential component of a healthy diet and the presence of a specific transporter (SVCT2) allows its internalization in neurons reaching a concentration 200-fold greater than in blood [4]. AA is readily oxidized to dehydroascorbate (DHAA) that can undergo irreversible hydrolysis to 2,3-diketo-l-gulonic acid, but because of its crucial role in CNS, DHAA is readily reconverted to AA to prevent vitamin C depletion. AA is also implicated in the protection against the excitotoxicity associated with high glutamate extracellular concentration through ascorbate/glutamate hetero-exchange [5,6]. Brain AA levels can be monitored amperometrically, using a carbon electrode poised at a mild anodic applied potential [5]:

(1) $$L-\text{Ascorbic Acid}\to \text{DHAA}+{2e}^{-}+{2H}^{+}$$

Oxygen, an essential molecule for life, is utilized not only for cellular respiration but also for biosynthesis and metabolism of various important biomolecules such steroids, eicosanoids, and neuroactive substances [7]. Oxygen is also implicated in several biochemical reactions involving for instance ATP in the brain [8]. Monitoring oxygen concentration dynamics could give important information about brain energy metabolism related to glucose [9] or lactate consumption [10]. The two-step electrochemical reduction of oxygen can achieved through amperometrically at a carbon-epoxy sensor surface as follows [8]:

(2) $$O_{2}+{2H}^{+}+{2e}^{-}\to H_{2}O_{2}$$

(3) $$H_{2}O_{2}\;+\;{2H}^{+}\;+\;{2e}^{-}\to 2H_{2}O$$

Glucose is actively involved in ATP synthesis and its concentration in extracellular spaces is the most important factor for energy metabolism [9,11,12]. Glucose detection is possible by means of a glucose oxidase (GOx)-based biosensor. GOx is covalently linked with flavin adenine dinucleotide (FAD) [13] and is extremely reliable because of its good sensitivity to the enzyme substrate and high stability when immobilized on Pt electrodes by means of poly-orthophenylenediamine (pOPD) [11,14]. Reactions occur as follows:

(4) $$\beta \text{-D-glucose}+{\text{FAD}}^{+}\text{-oxidase}\to \text{D-glucono-}\delta \text{-lactone}+{\text{FADH}}_{2}\text{-oxidase}$$

(5) $${\text{FADH}}_{2}\text{-oxidase}+O_{2}\to \text{FAD-oxidase}+H_{2}O_{2}$$

By applying a positive potential of 700 mV to the Pt working electrode, *versus* a Ag/AgCl reference electrode, the electrochemical oxidation of hydrogen peroxide occurs as follows:

(6) $$H_{2}O_{2}\;\to O_{2}+{2e}^{-}+{2H}^{+}$$

where the current produced by (6) is proportional to the concentration of glucose transformed by the enzyme.

Nowadays the most frequent use for biotelemetry is in medicine, in cardiac care units or step-down units in hospitals [15,16,17]. In this study, we present a wireless device connected to microsensors or biosensors capable of detecting rapid changes of AA, O_2_ and Glucose concentrations in the striatum of untethered freely-moving rats. The intrinsic chemical characteristics of these molecules allow their detection using specific telemetric devices able to work in oxidation [18,19] or in reduction [8] mode.

## 2. Results and Discussion

### 2.1. Biotelemetric device test and calibration

The electronic circuit of the miniaturized biotelemetric device was composed by three different parts: the amperometric module, the microcontroller and the transmitter. As described in Section 3.2 below, the amperometric module was made using three “rail-to-rail” operational amplifiers working as potentiostat (OPA1), voltage follower (OPA2) and current-to-voltage (I/V) converter (OPA3). The Zener diode (Z) plays a pivotal role in the amperometric circuitry generating a fixed voltage of 1.22 V useful for the fine regulation of the potential applied (V_App_) to the working electrode by means of a miniaturized potentiometer (P). The non-inverting input of OPA1 can be alternatively grounded or connected to Z for working in oxidation or reduction mode respectively. The transfer function of the I/V converter is:

(7) $$V_{\text{Out}}=-(I_{\text{redox}}\cdot R_{f})+V_{\text{App}}$$

in which I_redox_ is the current flowing through the WE, R_f_ is the feedback resistor and V_App_ is the potential applied to the WE. R_f_ has a capacitor in parallel (C_f_) to complete a low pass filter with a cut-off frequency (F_cut-off_) of 25 Hz. The value of C_f_ was calculated in farads according to the equation:

(8) $$C_{f}=1/(F_{\text{cut-off}}\cdot 2\pi \cdot R_{f})$$

An automated dummy cell was made based on a previously published design [8,20] for testing the amperometric module of the biotelemetric device. The calibration of the electronics was made indoors with a linear distance between the TX and RX units of about 3 m confirming previously-published results [8]. The averaged power consumption necessary to drive the biotelemetric device was experimentally determined [8,20,21] as 375 μW (125 μA). This means that a 3 V lithium coin battery (Maxell CR1216), having a capacity of 25 mA h^−1^, can power the unit for more than one week of continuous operation (sample rate: 1 Hz). The current necessary to drive the receiver unit was equal to 45 mA (225 mW). The biotelemetric device is characterized by gain precision, stability and an excellent linear response. The system can operate both in oxidation and reduction modes and it is particularly suited to work with direct-oxidation sensors (AA) or biosensors based on oxidase enzymes (glucose) and direct-reduction sensors (O_2_) or O_2_-consuming biosensors [22]. The weight of the biotelemetry unit is compatible with similar commercial devices [23], represents ∼ 3% of the rat body weight and it is well tolerated by the animals in agreement with other studies [24].

### 2.2. In-vitro calibration of ascorbic acid microsensor and in-vivo results

*In-vitro* calibrations of AA microsensors were carried out in fresh PBS at room temperature (25 °C) before and after implantation. A constant potential of +120 mV vs Ag/AgCl was applied and, after a stable baseline was reached, known amount of AA stock solution were added to the PBS in order to obtain concentrations ranging from 0 to 1 mM.

**Figure 1.**
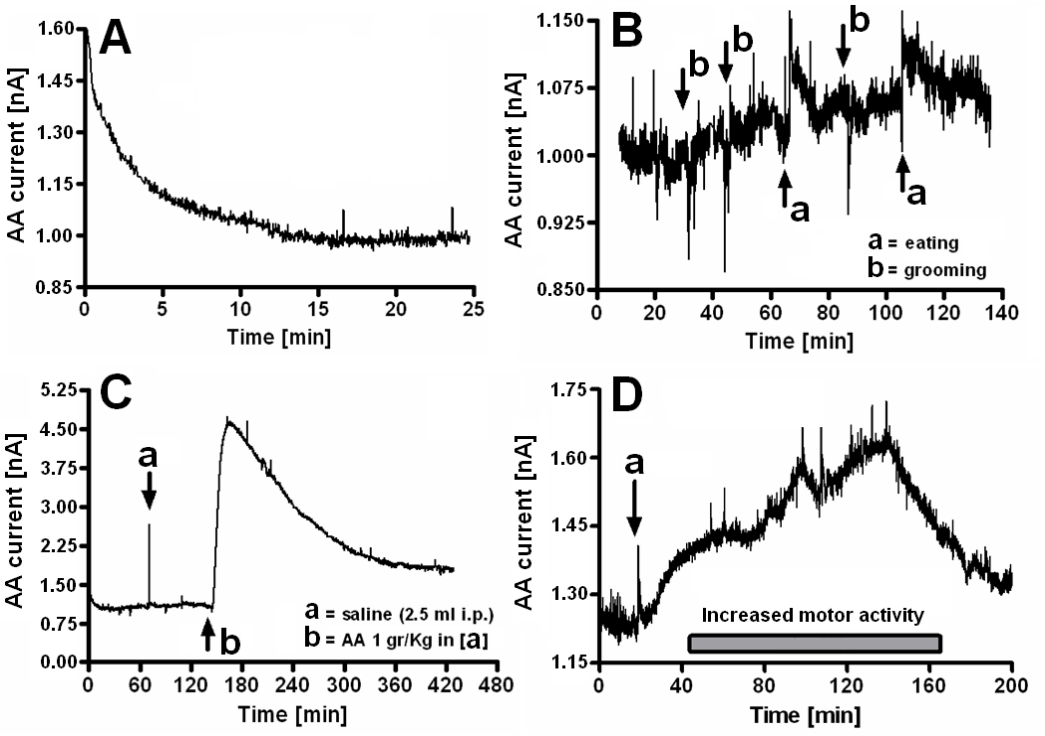
Physiological and pharmacologically-induced changes of striatal ascorbic acid. (A) AA microsensor *in-vivo* stabilization. (B) Striatal AA physiological changes during eating (a) and grooming (b). (C) Effect of saline (a) and sodium ascorbate (b) (1 gr kg^−1^ i.p. administration) on striatal AA current. (D) Effect of d-amphetamine (2 mg kg^−1^ s.c. administration) (a) on striatal AA current and motor activity.

Before implantation, microsensors showed good sensitivity and good linearity (7.3 pA μM^−1^, R^2^ = 0.9959). Post-implantation sensitivity dropped by about 60% (2.9 pA μM^−1^), but maintained a good linearity (R^2^ = 0.9959). *In-vivo* experiments started 24 h after implantation. A stable baseline was reached after a period of about 20 min (see Figure 1A). The calculated AA baseline corresponded to a concentration of ∼ 350 μM, in agreement with previous findings [25]. Physiological fluctuations of AA current were observed in concomitance with stereotyped behaviors (see Figure 1B). Pharmacological treatments were performed by administering sodium ascorbate (1 gr kg^−1^ i.p.) and d-amphetamine (2 mg kg^−1^ s.c.). Sodium ascorbate was administered intraperitoneally in order to verify sensor response and resulted in a 4-fold increase in striatal AA current (see Figure 1C). Subcutaneous d-amphetamine (see Figure 1D) induced an increase in AA current (+0.40 nA corresponding to +138 μM) and motor activity in accord with previous studies [26]. d-Amphetamine has also been shown to decrease glutamate striatal concentrations [26]. These findings are consistent with the functioning of an AA/glutamate heteroexchange system [6,27] in which AA release is linked to impulse traffic, transmitter release and glutamate uptake [26].

### 2.3. In-vitro calibration of oxygen microsensor and in-vivo results

All *in-vitro* calibrations of oxygen microsensors were carried out 24 h after manufacture, immediately before implantation and then repeated after *in-vivo* experiments, using a previously-described electrochemical cell [20,21], appropriately set for oxygen [8].

**Figure 2.**
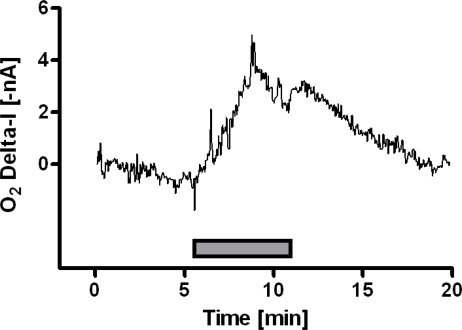
Effect of physiological stimulation on striatal dissolved oxygen. A 5-min tail pinch was applied to untethered, freely-moving rats resulting in an enhancement in motor and chewing activities, with an onset of a few seconds after the paper clip application, and a concomitant increase in striatal O_2_ current. Delta-I: inverted baseline-subtracted current.

The calibration performed before implantation exhibited good linearity with a slope of 213 ± 2 pA μM^−1^ of O_2_ (R^2^ = 0.989; n = 6), whilst the calibration made after implantation showed a reduction in sensitivity against O_2_ (−18%), in line with previous observations [8]. *In-vivo* experiments started 24 h after implantation. A stable baseline (19.7 ± 3.2 nA; n = 6) was reached after a period of about 45 min. Considering that the averaged background current of the microsensor in nitrogen-saturated PBS (day 0 and day 8) was around 14 nA, it is possible to estimate the concentration of O_2_ using *in-vitro* pre- and post-calibrations; this was found to correspond to 33 ± 14 μM, a value consistent with previous estimates. [28,29,30–32]. Physiological stimulation, a 5 min-tail pinch (see Figure 2), administered in order to increase neural activity and to promote regional cerebral blood flow (rCBF), led to increased motor activity and striatal O_2_ current of +4.8 nA, corresponding to +27 μM. Striatal oxygen dynamics, following physiological stimulation, results in a rise in the local O_2_ signal [8], mainly related to an increase of rCBF during neural activation in agreement with previous reports on wired rats [8,28,29].

### 2.4. In-vitro calibration of glucose biosensor and in-vivo results

**Figure 3.**
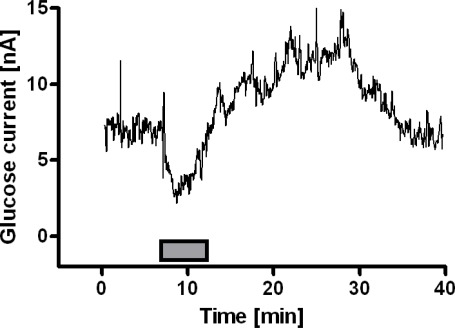
Effect of physiological stimulation on striatal glucose current. A 5-min tail pinch was applied to untethered, freely-moving rats resulting in an enhancement in motor and chewing activities with a concomitant decrease of glucose signal during stimulus administration followed by an increase of glucose current which then returned to baseline.

The *in-vitro* response of the glucose biosensor was determined just before implantation by adding known amounts of glucose in the electrochemical cell giving concentrations ranging between 0 and 140 mM. Calibrations showed classical Michaelis-Menten kinetics (R^2^ = 0.989, n = 6) with V_max_ and K_M_ equal respectively to 89 ± 4 nA and 4.8 ± 0.6 mM. The linear region was evaluated at low concentrations (0 – 2 mM), which showed good linearity (R^2^ = 0.987, n = 6) with a slope of 15.2 ± 1.1 nA mM^−1^. The *in-vivo* experiments were carried out using the same procedures as oxygen studies. A stable baseline was observed 30 – 35 min after sensor polarization and corresponded to 7.5 ± 0.5 nA (492 ± 35 μM from the above *in-vitro* calibration), in agreement with previous findings [11]. A 5-min tail pinch (see Figure 3) resulted in an initial decrease of glucose signal during stimulus administration, followed by an increase of glucose current which then returned to baseline after ∼ 30 min. These results are suggestive of glucose consumption during neural activation followed by an increase of extracellular levels of glucose, possibly due to astroglial glycogenolysis [33].

## 3. Experimental Section

### 3.1. Reagents, solutions and electronic parts

All chemicals were analytical reagent grade or higher purity and dissolved in bidistilled deionized water (MilliQ^®^). Ascorbic acid, sodium ascorbate, uric acid, dopamine, 3,4-dihydroxyphenylacetic acid (DOPAC), d-(+)-glucose, glucose oxidase from *Aspergillus Niger* (EC 1.1.3.4), *o*-phenylenediamine (OPD), Nafion^®^ (5% in aliphatic alcohols) and d-amphetamine were purchased from Sigma-Aldrich (Milano, Italy). The phosphate-buffered saline (PBS, 20 mM) solution was made using 0.15 M NaCl, 0.04 M NaH_2_PO_4_ and 0.04 M NaOH from Sigma, and then adjusted to pH 7.4. GOx solution was prepared by dissolving 180 units of enzyme in 10 μL of PBS and stored at −30 °C. The OPD monomer (250 mM) was dissolved in deoxygenated PBS immediately before use. Stock solutions of AA (100 mM) were prepared daily in water immediately before use, while the stock solution of glucose (1 M) was prepared in water as previously described [21]. Solutions were kept at 4 °C when not in use. Ultrapure (> 99.9%) oxygen (O_2_) and nitrogen (N_2_) were acquired from Sapio s.r.l Special Gases Division (Caponago, Italy). N_2_-purged and O_2_-saturated solutions, used for *in-vitro* calibrations, were obtained by bubbling the corresponding gas in 10 mL of PBS for 60 min. The air-saturated solution of PBS (21% O_2_) was obtained by dissolving filtered air in 10 mL of PBS for 1 h, using a diaphragm air pump. All *in-vitro* calibrations of oxygen microsensors were performed using freshly-prepared N_2_ and O_2_ solutions under standard conditions of pressure and temperature [8]. Electronic parts were from Farnell-In-One spa (Milano, Italy), the radio modules were from Telecontrolli spa (TC, Casoria, Italy) and the USB components preassembled by Futura Elettronica srl (Gallarate, Italy).

### 3.2. Biotelemetric device

**Figure 4.**
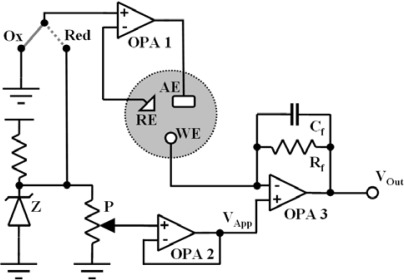
Amperometric section of the biotelemetric device. Ox: Oxidation; Red: Reduction; Z: Zener diode; P: Potentiometer; OPA: Operational Amplifier; R_f_: Feedback Resistor; C_f_: Feedback Capacitor; V_App_: Applied Potential; V_Out_: Output Voltage; WE: Working Electrode; RE: Reference Electrode; AE: Auxiliary Electrode.

The amperometric section of the biotelemetric device (see Figure 4), weighing less than 10 grams, was built using a quad single-supply operational amplifier MCP6044 (Arizona Microchip, Chandler, AZ, USA) and a ZXRE4001 Zener diode (Zetex, Manchester, UK). The ADC was an integral part of the microcontroller (PIC12F683, Arizona Microchip) used in this system. The 433.92 MHz AM transmitter was a RT4-433.92 (TC) while the RR3-433.92 module (TC) was selected as the AM receiver. Both TC modules were equipped with external antennas. The serial-to-USB converter was a FTDI-FT232R module with internal E_2_PROM and integrated clock synthesizer. All capacitors were NP0-type multilayer ceramic (low pass filter, decoupling) or electrolytic (decoupling). All resistors were precision metal oxide thick film (250 mW, 0.1% tolerance, Ohmite, Rolling Meadows, IL, USA). The components were soldered on single side PCB boards produced as previously described [8]. All electronic parts used in this project were Pb-free and compliant to RoHS directives. The biotelemetric device was derived from previous designs [8,20,21] and is capable of working in both oxidation (Ox) and reduction (Red) modes.

### 3.3. Preparation and calibration of microsensors and biosensors

**Figure 5.**
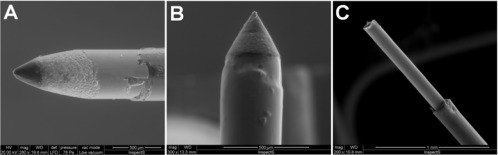
Scanning Electron Microscopy (SEM) microphotographs of (A) ascorbic acid microsensor, (B) oxygen microsensor and (C) glucose biosensor used in this study.

The AA microsensors were made using Teflon™-insulated silver wires (30 mm in length; Ø = 125 μm, Advent Research Materials, Suffolk, UK) modifying a previously-described procedure [34]. Approximately 1 mm of the wire was exposed and inserted into a silica capillary tube (10 mm in length; I.D. Ø = 180 μm, Polymicro Technologies, Phoenix, AZ, USA) partly filled with graphite-loaded (55% w/w) epoxy resin (Araldite-M®, Sigma-Aldrich, Milan, Italy). A preliminary 180 μm diameter carbon-composite disc electrode (area: 2.5 × 10^−4^ cm^2^) was fabricated by mixing 850 mg of graphite with 500 mg of Araldite-M and 200 mg of hardener and filling the silica capillary tubing with the mixture. The silver wire guaranteed a good electrical contact. After 24 h at 40 °C, the shape of the WE was transformed from disc to conical (see Figure 5A) using a high speed drill (Dremel^®^ 300) equipped with an aluminum oxide grinding wheel. The final AA microsensors had a length ≈ 250 μm, a surface area ≈ 1.5 × 10^−3^ cm^2^ and a tip diameter < 25 μm, dimensions well below those associated with significant tissue trauma caused by the implantation of larger probes [8,35,36]. The O_2_ microsensors (see Figure 5B) were manufactured in the same way as AA ones performing a further surface treatment with cellulose nitrate [8].

AA oxidation and O_2_ reduction potentials were experimentally established using cyclic voltammetry and were found to be +120 mV [19] and −400 mV [8], respectively vs Ag/AgCl (NaCl 3 M; RE4 Bioanalytical Systems, Inc., Lafayette, TX, USA) reference electrode. The fabrication of the glucose biosensors (see Figure 5C) has been previously described in detail [21]. Briefly, 1 mm Pt cylinder, obtained by cutting Teflon-insulated Pt wire (Ø = 125 μm, Advent Research Materials, Suffolk, UK), was immersed 3 times into a solution of GOx and let it dry for 5 min after each dip. The biosensor was then placed in the cell filled with 5 mL of N_2_-purged PBS containing the o-phenylenediamine monomer (250 mM). The electrosynthesis of p-OPD was carried out at +700 mV vs. Ag/AgCl for 15 min. H_2_O_2_ electro-oxidation was carried out at +700 mV [21] vs Ag/AgCl reference electrode. Constant potential amperometry (CPA) was used for *in-vitro* and *in-vivo* experiments; all *in-vitro* calibrations were performed in fresh PBS 24 h after sensors’ fabrication as previously described in detail [8,19,21]. No significant interference signals were observed on exposing AA, O_2_ microsensors and glucose biosensors to other electroactive molecules present in the striatal extracellular fluid (ECF), even at pharmacologically relevant concentrations [37] (Table 1).

### 3.4. Animals, stereotaxic surgery and in-vivo experimental procedures

Male Wistar rats (Morini R. Emilia, Italy), weighing 250 – 300 g were used in all experiments. Rats were kept under standard animal care conditions with 12 h light/dark cycle, and room temperature 21 °C, food and water *ad libitum*. Before each experiment, the health of the animals was assessed according to published guidelines [38]. All procedures were licensed under the European Community directive 86/609 included in Decreto No. 116/1992 of the Italian Ministry of Public Health. Stereotaxic surgery was performed under chloral hydrate (400 mg kg^−1^ i.p.) anesthesia. Microsensors and biosensors were implanted in the right striatum using the following coordinates from the atlas of Paxinos and Watson [39]: A/P +0.5 from bregma, +2.5 M/L, and −4.0 D/V from dura. Reference and auxiliary electrodes were implanted in the left parietal cortex and two screws were inserted in the skull. The biotelemetric device was fixed as previously described [8]. Body temperature during anesthesia was maintained at 37 °C by means of an isothermal heating pad. Following surgery, the animals were housed in large plastic bowls (45 cm diameter), and maintained in a temperature- and light- controlled environment, with free access to food and water. The sensors were polarized 24 h after surgery (day 1). The neurochemical monitoring started with the animal in its home bowl: this arrangement allowed the rat free movement. Physiological stimulation (tail pinch) and pharmacological treatments (sodium ascorbate and d-amphetamine) were carried out within the first week after stereotaxic surgery.

### 3.5. Hystology

After each set of experiments (day 8), rats were sacrificed with an injection of chloral hydrate (800 mg kg^−1^ i.p.). The location of each microsensor and biosensor in the striatum was confirmed by *post-mortem* histology. Brains were fixed in formal saline and 50 μm coronal sections were made with a cryostat. The slices were stained with cresyl violet and examined under a microscope.

### 3.6. Statistical analysis

Concentrations of AA, O_2_ and glucose were expressed in μM. AA and glucose (H_2_O_2_) anodic signals were given as absolute current values (nA) while oxygen cathodic current was expressed in nA and given as baseline-subtracted (Delta-I) raw data. The sign of the oxygen currents was inverted to give a positive correlation of the plotted data with the concentration of analyte. The *in-vitro* response of AA and oxygen microsensors was evaluated before and after in *in-vivo* experiments while the glucose biosensors parameters were calculated only before implantation because of the damage during explant can lead to inaccurate calibration [40]. The changes of brain tissue neurochemicals were calculated as absolute variations *versus* the corresponding baselines and their striatal concentrations were estimated using pre-implantation (glucose) or post-implantation (AA and O_2_) *in-vitro* calibrations.

## 4. Conclusions

In this study we present the real-time monitoring of three brain neurochemical species (AA, O_2_ and glucose) in untethered, freely-moving rats using a biotelemetric device coupled with implantable sensors. The transmitter and the receiver units have been used for accurate transduction of the redox currents generated on the surface of these microsensors and biosensors, both *in vitro* and *in vivo*. The miniaturized biotelemetric device, composed by an amperometric module, a microcontroller and a transmitter, polarizes the sensor and sends sensor data to a receiving unit connected to a PC. The system electronics have been tested under different experimental conditions exhibiting low power consumption, high stability and good linear response. The *in-vivo* results confirmed previously-published observations on striatal AA, oxygen and glucose dynamics. This approach, based on simple and inexpensive components, could be used as a rapid and reliable model for studying the effects of different drugs on brain neurochemical systems.

## Figures and Tables

**Table 1.** Effects of some electroactive molecules (AA, DOPAC, UA and DA) present in the striatal ECF on the amperometric response of the AA, O_2_ and glucose sensors. N.D.: (response) not detected.

**Interference**	**AA microsensor (n = 4)**	**O_2_ microsensor (n = 4)**	**Glucose biosensor (n = 6)**
**AA** (500 μM)	3.65 ± 0.4 nA	N.D.	0.73 ± 0.2 nA
**DOPAC** (10 μM)	31 ± 6 pA	N.D.	22 ± 5 pA
**UA** (10 μM)	16 ± 7 pA	N.D.	27 ± 9 pA
**DA** (1 μM)	N.D.	N.D.	55 ± 11 pA
